# EndoC-βH3 pseudoislets are suitable for intraportal transplantation in diabetic mice

**DOI:** 10.1080/19382014.2024.2406041

**Published:** 2024-09-19

**Authors:** Mengmeng Zhou, Thomas Linn, Sebastian Friedrich Petry

**Affiliations:** Clinical Research Unit and Working Group Experimental Diabetology and Islet Cell Biology, Medical Clinic and Polyclinic III, Center of Internal Medicine, Justus Liebig University, Gießen, Germany

**Keywords:** EndoC, human, human-to-mouse, islet transplantation, pseudoislets, streptozotocin, type 1 diabetes mellitus

## Abstract

**Background:**

Islet or β-cell transplantation is a therapeutical approach to substitute the insulin-producing cells which are abolished in type 1 diabetes mellitus. The shortage of human islets as well as the complicated and costly isolation process limit the application of these techniques in daily clinical practice. EndoC-βH is a human β-cell line that readily forms aggregates termed pseudoislets, providing an alternative to primary human islets or β-cells.

**Methods:**

EndoC-βH3 cells were seeded and incubated to form pseudoislets. Their insulin secretion was analyzed by ELISA and compared with cell monolayers. Pseudoislets were transplanted into streptozotocin-treated NMRi nu/nu mice. Blood glucose was monitored before and after transplantation and compared with wild types. Grafts were analyzed by immunohistology.

**Results:**

This study shows that EndoC-βH cells are able to form pseudoislets by aggregation, leading to an enhanced glucose stimulated insulin secretion in vitro. These pseudoislets were then successfully transplanted into the livers of diabetic mice and produced insulin in vitro. Blood glucose levels of the streptozocin-treated recipient mice were significantly decreased when compared to pre-transplantation and matched the levels found in control mice.

**Conclusion:**

We suggest pseudoislets aggregated from EndoC-βH cells as a valuable and promising model for islet transplantation research.

## GRAPHICAL ABSTRACT

**Figure:**
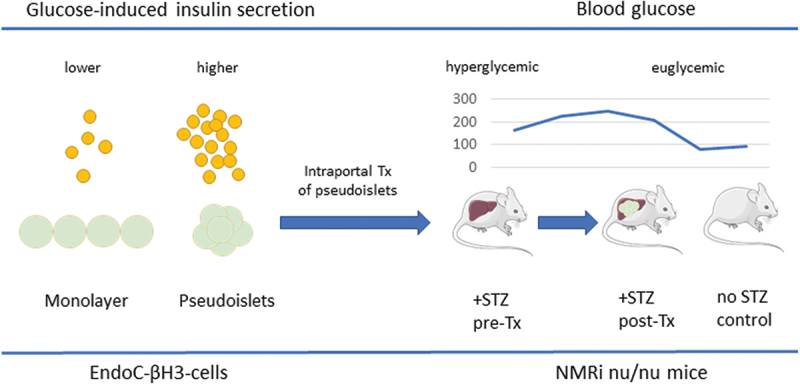

## 1. Introduction

Type 1 diabetes is caused by the autoimmune destruction of the insulin-secreting β-cells. The absolute lack of insulin requires patients to rely on the supplementation of exogenous insulin. However, it is difficult to achieve physiological control of blood glucose concentration, and chronic complications are still inevitable in many cases. Islet transplantation can replace exogenous insulin therapy and normalize metabolic control.^1^ Before the discovery of insulin, English surgeon Watson Williams made the first attempt to transplant sheep pancreas fragments into a 15-year-old boy suffering from ketoacidosis in 1893.^2^ Since then, islet isolation and transplantation technology have gradually progressed from research to clinical exercise. In 2022, the results of a single-center cohort study in Canada showed that among 255 patients who received islet transplantation between 1999 and 2019, 164 (64.3%) had transient and 87 (34.1%) sustainable graft survival.^3^ Although these results indicate promise, they also emphasize the need for future research.

Human islets differ from rodent islets in several ways, including endocrine cell distributions and arrangements,^4^ glucose set points,^5^ as well as transcriptomic, epigenomic, and chromatin signatures.^6^ To better understand and improve the efficacy of islet transplantation, research on human islets is necessary. However, the source of primary human islets is limited, and the isolation process is complicated and time-consuming. Therefore, pseudoislets formed by aggregation of human β-cells have become an alternative method to study human islets.

In the past, there have been numerous efforts to obtain a human β-cell line exhibiting a distinct glucose stimulated insulin secretion (GSIS). In 2011, the Scharfmann group described the EndoC-βH1 cell line as a genetically engineered human β-cell line obtained by targeted oncogenesis in human fetal pancreatic tissue.^7^ Despite their ability to glucose-dependently secrete insulin, there were marked differences of the EndoC-βH1-cells when compared to native human β-cells. Most importantly, EndoC-βH1-cells continue to proliferate. The excision of the immortalizing transgenes by a cre-mediated approach led to the EndoC-βH2 cell line. It was described as even more similar to the human β-cell with an improved insulin secretion.^8^ The EndoC-βH3 cell line is a new conditionally immortalized human β-cell line, in which the immortalizing transgene can be efficiently removed by simple addition of tamoxifen.^9^ When compared to its predecessor, the EndoC-βH3 cell line can be cleared of its immortalizing transgenes by treatment with tamoxifen and puromycin. It is marked by a 12-fold increase in insulin expression and a 23-fold increase in insulin content. Since these cells can be kept stable in culture for five weeks, they are described as being close to genuine human β-cells.^9^

It has been shown that pseudoislets formed by aggregation of EndoC-βH3 cells have increased insulin expression and GSIS *in vitro* compared with isolated cells,^10^ but little is known *in vivo*.

Therefore, in this study, the pseudoislets formed by the aggregation of EndoC-βH3 cells were inspected for mechanical robustness and operational functionality at transplantation via the intraportal route in laboratory mice.

## 2. Materials and methods

### 2.1. EndoC-βH cells and cell culture

EndoC-βH3 cells were cultured in Optiβ1 medium (Human cell design) onto βcoat-treated (Human cell design) culture flasks or plates. Each vial of βcoat was diluted in 10 ml Dulbecco’s Modified Eagle Medium (DMEM, Gibco) and substituted with 100 µl penicillin/streptomycin (Life Technologies). The culture conditions were 37°C and 5% CO_2_. Cells were split weekly by trypsinization with 0.05% trypsin – EDTA (Gibco) after washing with PBS (Gibco). The enzymatic reaction was stopped with DMEM (Gibco) containing 20% heat-inactivated fetal calf serum (biowest, Nuaillé, France). Cells were centrifuged (500 g for 5 min at room temperature) and resuspended with Optiβ1 medium. Cells were counted using a hemocytometer. Afterward, they were seeded at the density of 70,000/cm^2^. 10 µg/ml puromycin (InvivoGen) was added to the medium for 14 days to select the puromycin-resistant EndoC-βH3 cells, and to allow a better effect of excision of the immortalizing gene. 5 µg/ml puromycin was maintained in the subsequent culture. Inducible excision of Cre-mediated immortalizing transgenes was performed with the addition of 1 µM 4-hydroxytamoxifen (TAM) (Sigma-Aldrich) for 21 days. Thereafter, TAM was removed from the medium, and all experiments were conducted within the following 14 days employing non-proliferating cells excised by TAM.

### 2.2. Pseudoislets formation

**Figure 1.**
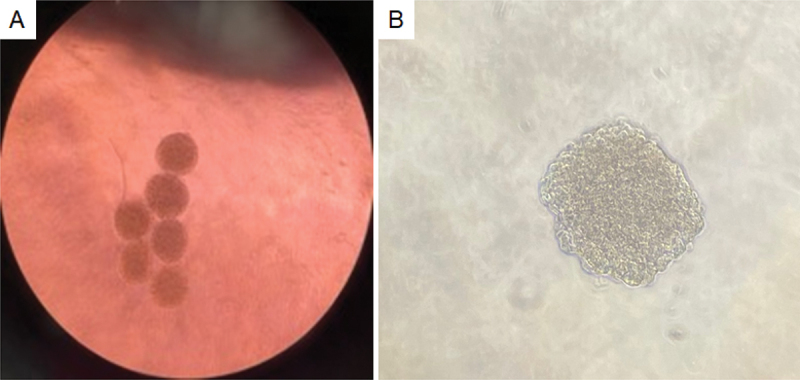
Representative images of pseudoislets formed after 48 h. (A) Six pseudoislets collected in a well after 48 h. (B) Higher magnification of a single pseudoislet.

EndoC-βH3 cells were seeded at a density of 2,000 cells/well onto Biofloat 96-well, U-bottom shaped plates (faCellitate, Germany) without coating after the 21 day-TAM treatment. Cells were incubated for 48–60 h in 100 µl/well Optiβ1 medium with 5 µg/ml puromycin. One pseudoislet was formed per well. They were carefully inspected and removed if they were irregularly formed. Also, cells which did not form a pseudoislet were disposed of. Six pseudoislets were used as one biological replicate of the respective conditions of the conducted experiments (Figure 1).

### 2.3. Insulin secretion and content

For GSIS analysis, the excised EndoC-βH3 cells were seeded onto βcoat-coated 6-well plates at a density of 5 × 10^5^ cells/well for 24 h. Pseudoislets were seeded for at least 48 h. Then monolayer cells and pseudoislets were incubated overnight in Optiβ2 medium (Human cell design) at 37 ^◦^C and 5% CO_2_ and saturating humidity followed by 60 min pre-incubation in βKREBS buffer (Human cell design) with 0.2% bovine serum albumin (BSA). The Optiβ2 medium was replaced by 0 mm and 20 mm glucose with or without 45 µM IBMX (Sigma-Aldrich) diluted in βKREBS buffer with BSA after 60 min of pre-incubation.

Cells or pseudoislets were lysed directly in the culture wells with TETG solution 20 mm Tris pH 8.0; 0.1% Triton X-100; 1% Glycerol; 137 mm NaCl; 2 mm EGTA and anti-protease tablet (Roche) for 5 min on ice for insulin measurement. Lysates were next centrifuged at 700 g for 5 min and stored at 20°C. Insulin secretion and intracellular content were measured in duplicate by enzyme linked immunoassay according to the manufacturer’s instructions (Mercodia).

### 2.4. Research animals

5 NMRi nu/nu mice were acquired at the age of 10 weeks from Charles River (Sulzfeld, Germany). The animals were given two weeks to adapt to the local animal facility. They were housed at 24 ± 2°C with a 14:10 h light/dark cycle, air exchange 15/h, relative humidity of 55 ± 10%, and provided with tap water and standard diet food ad libitum in individually ventilated cages in groups of 2–3 mice. All animal research was carried out in accordance with recommendations from our institutional animal welfare officer, the Chair of Animal Welfare of the Justus Liebig University, Giessen, and the Regional Administrative Council of Giessen, the Veterinary Department (GI 20/11-Nr.G 31/2017). All experiments were performed in accordance with the German Animal Welfare Law.

### 2.5. Islet transplantation via the intraportal route

Diabetes was induced in 4 NMRi nu/nu mice by intraperitoneal injection of 200 mg/kg body weight of Streptozotocin (STZ). A non-fasting blood glucose of ≥200 mg/dL was used as the criterion for diabetes mellitus. Three mice were randomly selected as recipients of pseudoislets. Glucose levels were monitored with a glucometer (OneTouch Ultra 2, LifeScan, Düsseldorf, Germany) using blood samples collected by puncturing the tip of the tail.

For each mouse, 300 pseudoislets were handpicked into a 50 ml conical tube along with Optiβ1 medium after 60 h of incubation. The tube was kept in a vertical position to allow the pseudoislets to settle. Optiβ1 medium was replaced with 0.3 ml of Hank’s solution. About 0.1 ml P/FCS was added to 300 pseudoislets in a 1 ml syringe, the volume was reduced to 0.05 ml, and then 0.15 ml Ficoll was added. The syringe was maintained vertically throughout the process, and the pseudoislets settled at the cone of the syringe within 2–3 min.

Preparation of animals and islet transplantation was performed as previously described by our group.^11^ In summary, mice were anesthetized by intraperitoneal injection of ketamine (100 mg/kg body weight) and xylazine (20 mg/kg body weight). After performing laparotomy, the pancreas was located by moving the duodenum. The portal vein was found on the ventral side of the pancreas by pushing up on it. Index finger and thumb were used to expose the portal vein and to puncture it near the liver. The pseudoislets were injected slowly and steadily into the portal vein within 1 min under the inspection of a magnifying glass to avoid any leakage of pseudoislets. To stop bleeding, a sterile gauze was placed at the puncture site and pressure was applied with the index finger (penetration depth: 0.5–1 cm) for 6 min. Finally, the incision and abdominal wall were closed. The syringe was checked for any remaining pseudoislets after injection by filling it with 1 mL of P/FCS medium and flushing it into a fresh Petri dish. Islets were counted under a microscope. After the transplantation, the non-fasting blood glucose was measured daily.

### 2.6. Immunohistochemistry

Livers and pancreases were collected at the end of the experiment and fixed with Roti-Histofix 4% formaldehyde solution (Carl Roth, Karlsruhe, Germany) and stored at 4°C. Before embedding, organs were washed with 70% ethanol on a shaker overnight at room temperature. Slides with a thickness of 5–7 µm were created using a microtome (Reichert Jung 2030, Germany) and stored at room temperature. Before staining, paraffin was removed with terpenes (Roti-Histol, Roth, Karlsruhe, Germany) and a decreasing alcohol series. Insulin was detected by light microscopy, and islet morphology was studied. Slides were washed with Tris, blocked for 20 min with 1% goat serum and incubated overnight at 4°C in a wet chamber with primary antibodies (Insulin, AB_1605150, Bio-Rad, Feldkirchen, Germany, 1:100) diluted in TBS containing 0.3% Triton X-100 (0.3% PBST), respectively. The secondary antibody (Goat-anti-guinea pig FITC, AB_567039, Bio-Rad, Feldkirchen, Germany, 1:100) diluted in 5% mouse serum in Tris was applied for 1 h at room temperature. Afterward, the vector red substrate kit (Vector Laboratories) was used to visualize insulin. Slides without primary antibodies were used as negative controls. The hematoxylin-eosin (HE) staining was used for histological evaluation.

PDX-1 (AB_11212087, Merck, Taufkirchen, Germany, 1:20) and Ki-67 (AB_2756525, Proteintech, Martinsried, Germany, 1:200), respectively, were co-stained with insulin (AB_794668, ThermoFisher, Darmstadt, Germany, 1:50) for the qualitative assessment of their expression (secondary antibodies: Alexa Fluor 488 Donkey-anti-guinea pig, AB_2340472, Jackson ImmunorResearch, Ely, UK, 1:600; Alexa Fluor 658 Donkey-anti-rabbit, AB_2534017, ThermoFisher, Darmstadt, Germany, 1:600). For these immunofluorescence analyses, tissue slides were treated with NaOH 0.09 M for three minutes and washed with Tris prior to blocking as described above.

### 2.7. Statistical analysis

GraphPad Prism 9 (GraphPad Software, San Diego, CA, USA) was employed for the statistical analysis, which included an unpaired t-test or one- or two-way ANOVA with Tukey or Sidak multiple comparison tests as appropriate. Data are presented as mean values *±* SEM unless otherwise stated. A *p*-value of <.05 was considered significant.

## 3. Results

### 3.1. Aggregation of EndoC-βH3 cells increases glucose stimulated insulin secretion

**Figure 2.**
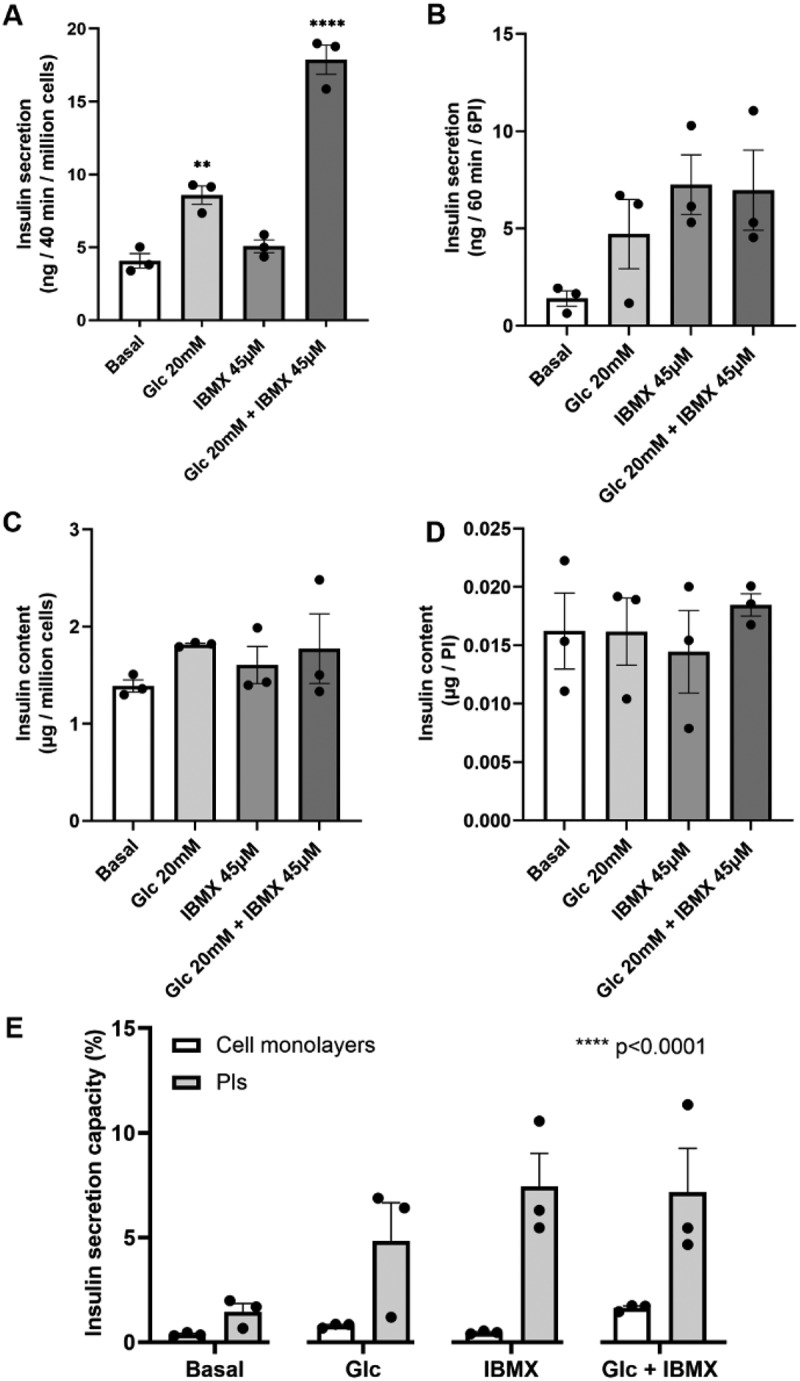
Glucose stimulated insulin secretion in EndoC-βH3 cell monolayers and pseudoislets. (A, B) glucose stimulated insulin secretion of EndoC-βH3 cell (A) monolayers and (B) pseudoislets. (C, D) insulin content of EndoC-βH3 cell (C) monolayers and (D) pseudoislets. (E) Insulin secretion capacity given as the proportion of insulin secreted into the incubation medium to the insulin content. *n* = 3, **** denotes *p* < .0001 and **p < .005 (one or two-way ANOVA).

To investigate their endocrine function, glucose stimulated insulin secretion was measured in EndoC-βH3 cell monolayers and pseudoislets, respectively. When cell monolayers and pseudoislets were exposed to 20 mM glucose, insulin secretion was stimulated 2.1- and 4.6-fold, respectively, indicating that the EndoC-βH3 cell is glucose-sensitive. In the presence of IBMX, insulin secretion was both stimulated 4.38-and 4.93-fold, respectively (Figure 2A,B). Insulin content did not change significantly in cells nor in pseudoislets (Figure 2C,D). Since the number of cells in pseudoislets and cell monolayers was inconsistent, we calculated the insulin secretion capacity based on insulin content and insulin secretion by dividing mean insulin secretion by mean basal insulin content (Figure 2E). Compared with cell monolayers, the insulin secretion capacity of pseudoislets was significantly increased. Taken together, these results indicate that insulin secretion is enhanced when cells aggregate in pseudoislets.

### 3.2. Engrafted pseudo islets are functional and stable in vivo

**Figure 3.**
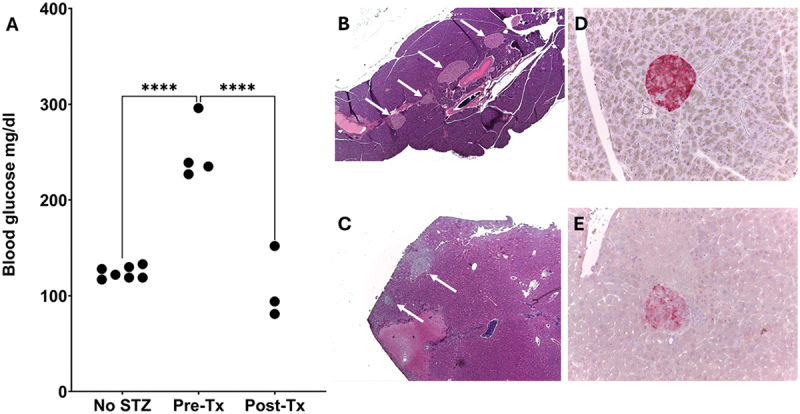
Blood glucose, morphology, and insulin staining of the pseudoislets and pancreatic islets of the pre/post-transplantation NMRi nu/nu mice. (A) Blood glucose of the pre/post-transplantation NMRi nu/nu mice with mice without STZ as control. Post-tx values were significantly lower than pre-tx. (B, C) Representative images of HE staining of the (B) pancreas and (C) liver. (D, E) Representative images of insulin staining of native (D) pancreatic islets and (E) transplanted pseudoislets in liver. White arrows indicate islets or pseudoislets, respectively. *n* = 3-7 mice, **** denotes *p* < .0001.

**Figure 4.**
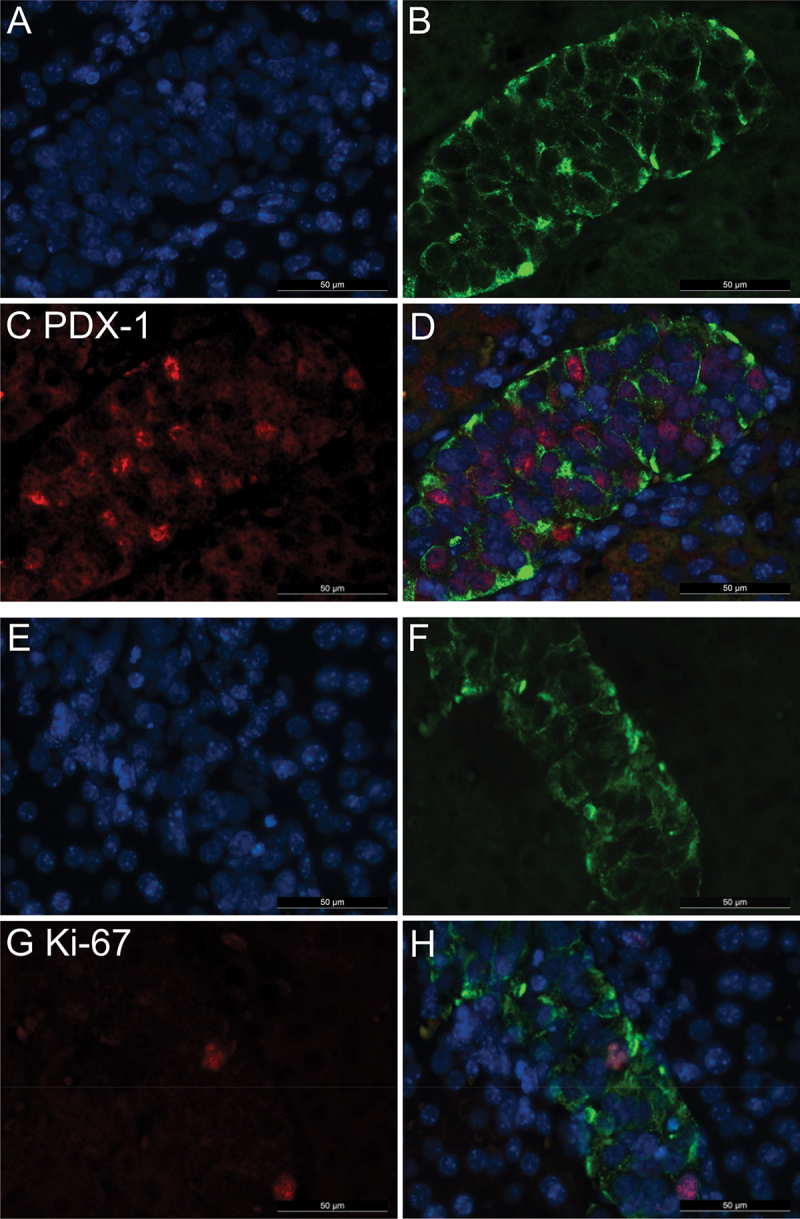
Immunohistological staining for PDX-1 and Ki-67 in transplanted pseudoislets. Representative images of pseudoislet grafts in NMRi nu/nu liver. (A) Nuclei (blue), (B) insulin (green), (C) PDX-1 (red) staining, and (D) the overlay; (E) nuclei (blue), (F) insulin (green), (G) Ki-67 (red) staining, and (D) the overlay. Images were taken at 400× magnification. The white bar represents 50 µm.

To investigate the potential of pseudoislets formed by Endoc-βH3 cells for intraportal islet transplantation, they were transplanted into diabetic mice. First, hyperglycemia was induced by STZ injection. Then the pseudoislets were injected with a 1 ml syringe via the intraportal vein following an abdominal cut. After transplantation, blood glucose and body weight were measured daily. Blood glucose was significantly decreased compared to pre-transplantation (Figure 3A), indicating satisfactory engraftment and favorable endocrine function. Five days after transplantation we collected livers and pancreas. Grafts located by HE staining (Figure 2C) were of similar morphology as native pancreatic islets (Figure 3B). Immunohistological insulin staining confirmed the grafts’ insulin content (Figure 3E), albeit staining intensity was lower in most grafts than in native pancreatic islets (Figure 3D). This was confirmed by immunohistology (Figure 4D,F). The immunohistological studies further revealed that the major beta-cell marker PDX-1 was still expressed strongly in pseudoislets after engraftment (Figure 4A-D). Ki-67 expression was occasionally present in a few cells of the engrafted pseudoislets (Figure 4E-H).

## 4. Discussion

The failure and decay of pancreatic β-cells is still inevitable in type 1 diabetes mellitus. Strategies aiming to replace the insulin-producing cells by transplantation are among the most important research approaches. The use of rodent cells to form pseudoislets as models for islet function and islet transplantation research has been described in many publications.^12–15^ EndoC-βH cells are a novel human β-cell line renowned for the formation of pseudoislets. These aggregates are comparable to human islets, providing a new model for islet transplantation research. Friedlander et al. summarize the established methods for human pseudoislet formation, including spontaneous reaggregation, microwell, and hanging drop.^13^ The authors conclude that pseudoislets are an excellent option for transplantation as they feature physiological functions of islets and offer exciting options for genomic editing. Cornell et al. have delivered evidence that cell–cell contacts regulate the metabolic flux and thereby improve the glucose-induced ATP-production, accounting for the increased GSIS observed in β-cell pseudoislets.^14^ In the present study, we seeded EndoC-βH3 cells on U-bottom shaped 96-well plates, and after 48 h leading to spontaneous aggregation into clusters. *In vitro*, these pseudoislets featured a significantly increased GSIS when compared with non-aggregated cells, consistent with the findings of Lecomte et al.^10^ Interestingly, these pseudoislets were engrafted successfully into the liver of diabetic mice by transplantation through the intraportal route. Although immunohistological staining showed a markedly lesser staining intensity when compared with native islets, the recipient mices’ blood glucose levels were significantly lower than before transplantation, indicating that pseudoislets formed by aggregation of EndoC-βH3 cells can not only be transplanted effectively into the liver through the intraportal route but also exhibit proper insulin secretion *in vivo*.

Analysis of PDX-1 revealed that pseudoislets maintain their β-cell identity during engraftment. Further, cell proliferation as indicated by Ki-67 is diminished but not completely abolished after tamoxifen treatment. As studied by Benazra et al., the excision of the transgene leads to a time-dependent arrest of cell proliferation as indicated by a significantly decreased Ki-67 mRNA expression.^9^ Thus, future studies will have to address the question of whether pseudoislets might continue to proliferate *in vivo* or even form malign tumors.

A wide range of publications has described EndoC-βH cells as close to genuine human insulin producing cells in terms of β-cell identity, i.e. GSIS,^9^ secretomes, transcriptome, and proteome,^16^ and electrophysiology.^17^

Interestingly, Lawlor et al. have described common signatures of gene expression, transcription factor binding, and cis-regulatory elements between EndoC-βH cells and primary human islets in a thorough multiomic profiling,^18^ indicating that the EndoC-βH-cell lines are excellent models to study β-cell physiology and pathophysiology. The suitability of EndoC-βH1-pseudoislets for human-to-mouse transplantation has been demonstrated by Tsonkova et al. The authors could confirm *in vivo* functionality, i.e. the responsiveness to cytokines, glucotoxicity and lipotoxicity.^19^ Our study confirms the feasibility of the transplantation of EndoC-βH-cell pseudoislets by employing the EndoC-βH3 cell line. Novel data generated by omics approaches and genome-wide screening might identify targets to improve graft survival and function.^20^ The latest available EndoCβH5-cell was described as being responsive to stimulation by glucagon-like peptide-1 and gastric inhibitory polypeptide. Further, it expresses the glucagon receptor and shows a homogenous β-cell identity providing an even more physiological behavior and a high level of experimental reproducibility.^21^ In summary, genetically engineered human β-cells offer exciting novel options for β-cell replacement therapies. According to the available literature, the formation of pseudoislets allows for an efficient and close to physiological creation of grafts. In the future, pseudoislets transplantation might even be enriched by the integration of other types of islet-cells which are of relevance to islet physiology.^22^

Although there are some limitations of our study, such as a small sample size, a short observation time, and a limited analysis of post-transplant pseudoislets, we conclude that the transplantation of human EndoC-βH cell pseudoislets is a technically feasible option for β-replacement and provides options for further transplantation experiments. In the future, long-term stability and function as well as graft rejection and tumorous potential have to be studied to develop respective transplantation protocols ensuring long-term graft viability and insulin production.
